# Characterization of *bla*_KPC-2_ and *bla*_NDM-1_ Plasmids of a *K. pneumoniae* ST11 Outbreak Clone

**DOI:** 10.3390/antibiotics12050926

**Published:** 2023-05-18

**Authors:** Camila Maria dos Santos Boralli, Julian Andres Paganini, Rodrigo Silva Meneses, Camila Pacheco Silveira Martins da Mata, Edna Marilea Meireles Leite, Anita C. Schürch, Fernanda L. Paganelli, Rob J. L. Willems, Ilana Lopes Baratella Cunha Camargo

**Affiliations:** 1 Laboratory of Molecular Epidemiology and Microbiology, Department of Physics and Interdisciplinary Science, São Carlos Institute of Physics, University of São Paulo, São Carlos 13563-120, Brazil; camila.boralli@usp.br; 2 University Medical Center Utrecht, Heidelberglaan 100, 3584 CX Utrecht, The Netherlands; j.a.paganini@umcutrecht.nl (J.A.P.); r.silvameneses@umcutrecht.nl (R.S.M.); a.c.schurch@umcutrecht.nl (A.C.S.); f.paganelli@umcutrecht.nl (F.L.P.); rwillems@umcutrecht.nl (R.J.L.W.); 3 Risoleta Tolentino Neves Hospital, Belo Horizonte 31744-012, Brazil; camila.mata@hrtn.fundep.ufmg.br (C.P.S.M.d.M.); edna.leite@hrtn.fundep.ufmg.br (E.M.M.L.)

**Keywords:** *bla*_KPC_, *bla*_NDM_, *Klebsiella pneumoniae*, plasmid, conjugation rate, plasmid copy number

## Abstract

The most common resistance mechanism to carbapenems is the production of carbapenemases. In 2021, the Pan American Health Organization warned of the emergence and increase in new carbapenemase combinations in *Enterobacterales* in Latin America. In this study, we characterized four *Klebsiella pneumoniae* isolates harboring *bla*_KPC_ and *bla*_NDM_ from an outbreak during the COVID-19 pandemic in a Brazilian hospital. We assessed their plasmids’ transference ability, fitness effects, and relative copy number in different hosts. The *K. pneumoniae* BHKPC93 and BHKPC104 strains were selected for whole genome sequencing (WGS) based on their pulsed-field gel electrophoresis profile. The WGS revealed that both isolates belong to ST11, and 20 resistance genes were identified in each isolate, including *bla*_KPC-2_ and *bla*_NDM-1_. The *bla*_KPC_ gene was present on a ~56 Kbp IncN plasmid and the *bla*_NDM-1_ gene on a ~102 Kbp IncC plasmid, along with five other resistance genes. Although the *bla*_NDM_ plasmid contained genes for conjugational transfer, only the *bla*_KPC_ plasmid conjugated to *E. coli* J53, without apparent fitness effects. The minimum inhibitory concentrations (MICs) of meropenem/imipenem against BHKPC93 and BHKPC104 were 128/64 and 256/128 mg/L, respectively. Although the meropenem and imipenem MICs against *E. coli* J53 transconjugants carrying the *bla*_KPC_ gene were 2 mg/L, this was a substantial increment in the MIC relative to the original J53 strain. The *bla*_KPC_ plasmid copy number was higher in *K. pneumoniae* BHKPC93 and BHKPC104 than in *E. coli* and higher than that of the *bla*_NDM_ plasmids. In conclusion, two ST11 *K. pneumoniae* isolates that were part of a hospital outbreak co-harbored *bla*_KPC-2_ and *bla*_NDM-1_. The *bla*_KPC_-harboring IncN plasmid has been circulating in this hospital since at least 2015, and its high copy number might have contributed to the conjugative transfer of this particular plasmid to an *E. coli* host. The observation that the *bla*_KPC_-containing plasmid had a lower copy number in this *E. coli* strain may explain why this plasmid did not confer phenotypic resistance against meropenem and imipenem.

## 1. Introduction

Carbapenems are a powerful group of broad-spectrum β-lactam antibiotics, which are often the antibiotics of last resort against multidrug-resistant bacterial infections [1,2,3]. The increased use of carbapenems because of the lack of treatment alternatives has triggered the selection of resistance to these antibiotics among an increasing number of pathogens, mainly due to the production of carbapenemases [2,4,5,6].

Carbapenemases are divided into two main types: metallo-β-lactamases (Class B), containing zinc in the enzyme’s active site, and serine β-lactamases (Classes A, C, and D), containing serine in the active site [7]. These enzymes have the highest degradation spectrum/potential among the β-lactamases, which are able to hydrolyze practically all β-lactams [2,8,9,10]. Although carbapenemase-encoding genes were first identified only on the chromosome; many are now transposon/plasmid-mediated, increasing the potential of horizontal transmission [11,12,13].

Currently, *Klebsiella pneumoniae* carbapenemase (KPC) is the most clinically significant serine carbapenemase in most countries in the world, and due to the fact of its rapid international spread, KPC threatens global public health [4,11,14]. In Brazil, reports indicate KPC as the main carbapenemase, with a prevalence of more than 76% among carbapenem-resistant *Enterobacterales,* while the New Delhi metallo-carbapenemase (NDM) is found at lower frequency and prevalence [15,16,17]. Since its first identification in Brazil, in 2013, NDM-producing bacteria have been considered Brazil’s next public health threat [17,18,19].

Latin America has seen the emergence and rise of novel combinations of carbapenemases in *Enterobacterales*, as well as the presence of carbapenemases that had not been previously described, according to a warning issued by the Pan American Health Organization. Argentina, Uruguay, Ecuador, Guatemala, and Paraguay identified isolates co-producing KPC and NDM with increasing rates between 2020 and 2021 compared to previous years, mainly in *K. pneumoniae* [20].

According to data from South and Midwest Brazil, the number of isolates co-producing NDM and KPC increased by almost six times during the COVID-19 pandemic, and they were detected mainly in the *Klebsiella pneumoniae* complex (59.5%), followed by *Pseudomonas aeruginosa* (12.9%) and *Serratia marcescens* (8.8%) [21]. The increase in KPC/NDM co-producers during the COVID-19 pandemic represents a worrying scenario for public health in Latin America [21,22,23]. In Brazil, ceftazidime–avibactam (CAZ-AVI) and aztreonam (ATM) are available to treat infections caused by KPC-producer and NDM-producer bacteria, respectively. However, these drugs alone are inefficient at treating infections with KPC/NDM co-producers. Some cases in the literature have shown the synergism and efficacy of treating infections using the combination of CAZ-AVI and ATM against serine- and metallo-β-lactamase co-producing *Enterobacterales* and *P. aeruginosa* [24,25]. A promising drug in these cases would be cefiderocol (CFDC), a novel siderophore cephalosporin, with activity against carbapenem-resistant bacteria, including multidrug-resistant (MDR) *Enterobacterales* and nonfermenters, due to the fact of its distinct penetration mechanism, which uses active iron transporters, and its stability against carbapenemases [26,27,28]. CFDC showed high activity against MBL-producing isolates and some dual-carbapenemases producers, such as KPC + VIM, NDM + OXA-48-like, and VIM + OXA-48-like [29,30]. However, evidence of activity against KPC/NDM co-producers is limited [29].

In this study, we molecularly characterized four clinical isolates from an outbreak of KPC/NDM co-producer *K. pneumoniae,* which started during the COVID-19 pandemic in a teaching hospital in Belo Horizonte, MG, Brazil. In addition, we sequenced two isolates and compared the identified *bla*_KPC_-carrying plasmid to the plasmids of a previous KPC producer isolate from the same hospital. Finally, we attempted to shed light on why the *bla*_KPC_ gene is usually more frequent in *K. pneumoniae* by studying the conjugation efficiency of the *bla*_KPC_-containing and *bla*_NDM_-containing plasmid to *E. coli* and assessing their copy number in both species.

## 2. Results

### 2.1. A Multidrug-Resistant K. pneumoniae ST11 Clone Spread during the COVID-19 Pandemic

In October 2020, a Brazilian teaching hospital (in Belo Horizonte/MG) detected the first KPC/NDM co-producer *K. pneumoniae*, which led to a molecular investigation of four isolates. According to the susceptibility profile, the four isolates involved in the outbreak are resistant to multiple antibiotics, and among all antibiotics that were tested, three of the four isolates were only susceptible to amikacin (Table 1). Isolate BHKPC107b was also susceptible to gentamicin.

**Figure 1:**
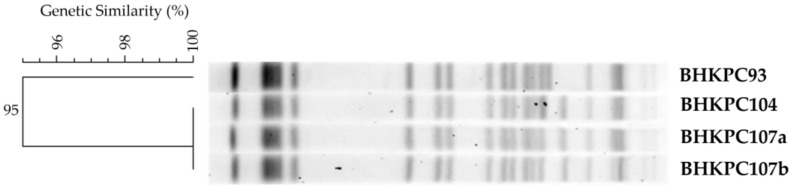
High genetic similarity of the four KPC and NDM co-producer isolates observed after XbaI digestion, pulsed-field gel electrophoresis, and analysis using Bionumerics software v. 7.1. The running direction of the gel was from left to right.

DNA macro-restriction followed by pulsed-field gel electrophoresis revealed the existence of one pulsotype, showing the clonal dissemination of KPC and NDM co-producer *K. pneumoniae* from the Brazilian hospital. The BHKPC93 strain had two differences, an additional band and the lack of another one, resulting in 95% genetic similarity, and was considered a closely related subtype (Figure 1). Therefore, BHKPC93 and BHKPC104, as representative isolates of two clonal types, were selected for whole genome sequencing (WGS) using Illumina and Nanopore technologies.

The nanopore mean coverage for BHKPC93 and BHKPC104 was 19×, and the Illumina’s were 134× and 106×, respectively. 

**Figure 2:**
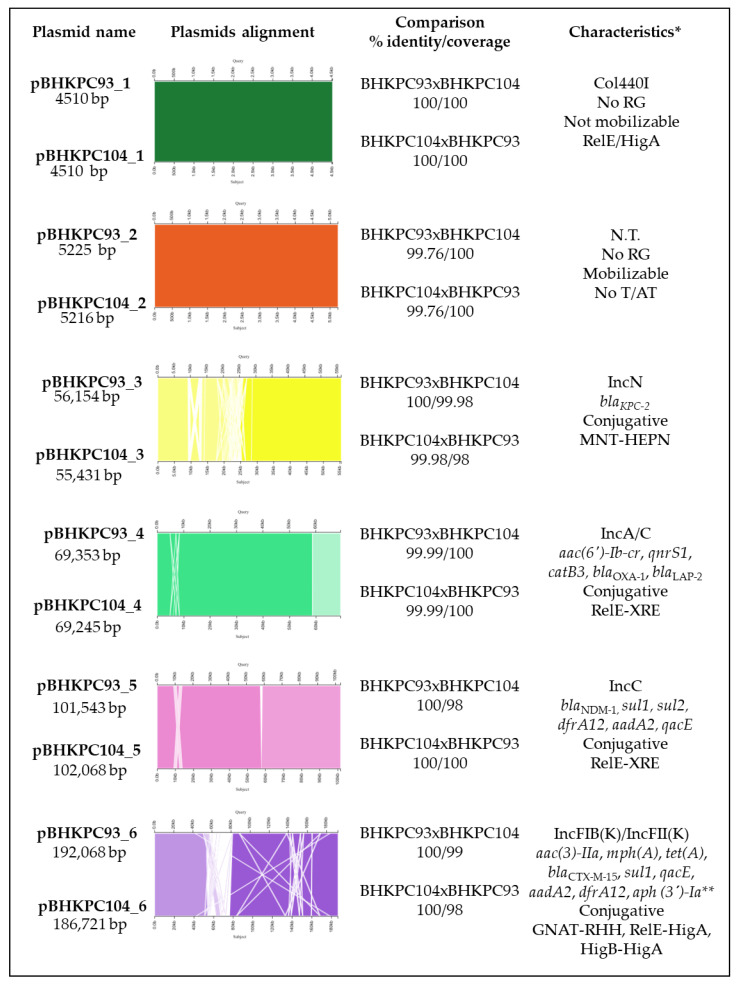
General information and comparison among the plasmids of the BHKPC93 and BHKPC104 strains. * Characteristics observed: plasmid incompatibility group; resistance genes; conjugation ability; toxin/antitoxin system. No RG, no resistance gene; N.T., non-typeable plasmid; No T/AT, no toxin/antitoxin system detected. ** Resistance gene present only in BHKPC104.

The assembly of the short-read and long-read sequencings of the two isolates, BHKPC93 and BHKPC104, resulted in a closed chromosomal sequence of BHKPC93 and BHKPC104 of 5,749,747 bp and 5,742,965 bp (Figure S1—Supplementary Materials), respectively, and six complete plasmid sequences with sizes ranging between 4.5 kbp and 193 kbp (Figure 2). 

We analyzed the similarity between the isolates BHKPC93 and BHKPC104 based on the gene content. We found that both isolates shared 5425 genes, while only 15 and 11 genes were exclusively found in BHKPC93 and BHKPC104, respectively; this resulted in a Jaccard distance of 0.0046 (Table S2—Supplementary Materials). Moreover, only 13 SNPs were found among shared genome fractions, thus confirming that the isolates were highly related.

The isolates belong to ST11 and harbor the *bla*_KPC-2_ and *bla*_NDM-1_ genes, plus 18 resistance genes. We found resistance genes to aminoglycosides (*aac(6’)-Ib-cr*, *aac(3)-IIa*, *aph(3’)-Ia*, and *aadA2*), fosfomycin (*fosA*), aminocyclitol (*aadA2*), quinolones (*aac(6’)-Ib-cr*, *oqxA*, *oqxB*, and *qnrS1*), folate pathway antagonists, such as trimethoprim-sulfamethoxazole (*sul1*, *sul2*, *dfrA12*, *oqxA*, and *oqxB*), tetracycline (*tet(A)*), β-lactams (*bla*_NDM-1_, *bla*_KPC-2_, *bla*_SHV-182_, *bla*_OXA-1_, *bla*_CTX-M-15_, and *bla*_LAP-2_), quaternary ammonium compounds (*oqxA*, *oqxB*, and *qacE*), amphenicol (*catB3*, *oqxA*, and *oqxB*), and macrolide (*mph(A)*) (Table S3—Supplementary Materials). The genes *fosA*, *oqxA*, *oqxB*, and *bla*_SHV-182_ were located on the chromosome, and the remaining genes were distributed on four plasmids (Figure 2). 

In addition, both isolates presented known mutations in the *gyrA* and *parC* genes, leading to the amino acid substitutions S83I and S80I, respectively, previously shown to result in ciprofloxacin resistance [31,32].

Next, we explored possible mechanisms of polymyxin resistance in BHKPC93 and BHKPC104. The two isolates did not contain *mcr* gene family sequences encoding acquired colistin resistance determinants. Additionally, we compared the sequences of the genes *mgrB*, *phoP*, *phoQ*, *pmrA*, *pmrB*, *pmrC*, *pmrK*, *pmrR*, *qceB*, *qceC*, *crrA*, and *crrB*, previously implicated in polymyxin resistance, of BHKPC93 and BHKPC104 with the polymyxin-susceptible ST11 *K. pneumoniae* AMKP36 strain [33] (genome accession number: SAMN13160271). This comparison did not reveal specific mutations in the two polymyxin-resistant isolates BHKPC93 and BHKPC104. 

BHKPC93 and BHKPC104 carried mutations in the *ompK36* and *ompK37* porin genes that potentially contribute to cephalosporin and carbapenem resistance and can also be involved in polymyxin resistance [34,35].

The string test classified BHKPC93, BHKPC104, BHKPC107a, and BHKPC107b as mucoid. The genome-sequenced strains also have seven virulence factors involved in heat resistance/thermal stress (*clpK1*), attachment to surfaces (*fimH* and *mrkA*), iron acquisition–siderophores genes (*fyuA*, *irp2*, and *iutA*), and serum resistance (*traT*).

### 2.2. The K. pneumoniae Isolates Have 16 Resistance Genes Distributed in Four Out of Their Six Plasmids

The BHKPC93 and BHKPC104 complete genome sequencing indicated that these isolates harbor six plasmids ranging from 4510 bp to 192,068 bp, which were very similar between the two strains (Figure 2).

pBHKPC93_1 and pBHKPC104_1 are 100% identical, with 4510 nucleotides (nt) Col440I-type plasmids that are predicted not to be mobilizable and harboring a RelE-like/HigA toxin/antitoxin system. 

pBHKPC93_2 and pBHKPC104_2 are non-self-conjugative but mobilizable plasmids that share 99.76% identity. Their incompatibility groups were not identified. In addition to the conjugation system genes, these plasmids harbor DNA methyltransferase, hypothetical protein, and replication initiation protein genes.

pBHKPC93_3 and pBHKPC104_3 are IncN *bla*_KPC_-harboring plasmids of 56,154 nt and 55,431 nt, respectively, sharing 99.98% identity. 

pBHKPC93_3 is larger than pBHKPC104_3 because it has a duplicated region composed of genes coding for two hypothetical proteins and the cobalamin biosynthesis protein gene (*cbiX*). In contrast, pBHKPC93_3 lacked the *adrA* gene that codes for an antirestriction protein, which was present in pBHKPC104_3. These plasmids carried relaxase genes codifying the T4CP and T4SS proteins, classifying them as conjugative plasmids. The *bla*_KPC_ gene is in a Tn*44011b* isoform inserted into a gene codifying a phospholipase D family protein in both plasmids (Figures S2 and S3—Supplementary Materials).

pBHKPC93_4 and pBHKPC104_4 are IncA/C-conjugative plasmids of 69,353 nt and 69,245 nt, respectively, carrying five resistance genes and the same toxin/antitoxin system as pBHKPC93_5 and p_BHKPC104_5.

pBHKPC93_5 and pBHKPC104_5 are IncC plasmids harboring *bla*_NDM_, which are 102,068 nt and 101,543 nt, respectively. In addition to *bla*_NDM_, these plasmids carry five other resistance genes: *sul1*, *sul2*, *dfrA12*, *aadA2*, and *qacE*. Although a relaxase gene was not identified using Plascad software, we used the Pfam database and found a gene annotated as a hypothetical protein that matches the MobI codifying gene. We also found genes encoding proteins involved in transfer by conjugation: type IV secretion system protein, TrbG/VirB9 family P-type conjugative transfer protein, conjugal transfer protein TraF, Conjugal transfer protein TraG, Conjugal transfer protein TraH genes. Based on the presence of these genes, these plasmids were classified as conjugative plasmids. The plasmids share 100% identity but 98% coverage due to the absence of an IS*Kpn18* gene in pBHKPC104_5. A ΔTn*125-4* harbors the *bla*_NDM_ gene with the bleomycin resistance gene (Figures S4 and S5—Supplementary Materials).

The largest plasmids, carrying eight resistance genes, with a complete conjugation system, are pBHKPC93_6 and pBHKPC104_6, with 100% identity and 99/98% coverage. In addition, pBHKPC104_6 has the *aph(3’)-Ia* gene and lacks the Tn*3*-like element Tn*5403* family transposase gene present in pBHKPC93_6. 

### 2.3. A Retrospective Search of bla_KPC_ Plasmids

Some of the bacteria from this hospital had already been sequenced before, and their genomes were deposited in GenBank. Among them, we identified the *K. pneumoniae* BHKPC49 strain (access number: SAMN32643894), which contained an IncN plasmid carrying *bla*_KPC_, which was already circulating in 2015. *K. pneumoniae* BHKPC49 belongs to ST147 and shares five virulence genes (*fimH, mrkA*, *fyuA*, *irp2,* and *iutA*) with strains BHKPC93 and BHKPC104, in addition to *ccl* and *nlpI*.

BHKPC49 harbors an IncN/IncR plasmid of 134 kbp, carrying *bla*_KPC_ in Tn*4401* and another 9 kbp ColRNAI plasmid. The *bla*_KPC_ plasmid of BHKPC49 contains the complete sequence of the IncN plasmids pBHKPC104_3 and pBHKPC93_3 and an IncR region harboring another 13 resistance genes: *aph(3″)-Ib*, *aph(6)-Id*, *aac(3)-IIa*, *aac(6′)-Ib-cr*, *qnrS1*, *dfrA1*, *sul1*, *tet(A)*, *bla*_OXA-1_, *bla*_TEM-1B_, *bla*_CTX-M-15_, *qacE*, and *catB3.* The BHKPC49 IncN/IncR plasmid shares the same toxin/antitoxin system (MNT-like/HEPN-like) as pBHKPC104_3 and pBHKPC93_3, and it has two other toxin/antitoxin systems (PIN-like/AbrB-like and Rel-like/Xre-like). The plasmid harboring the *bla*_KPC_ gene that circulated at the hospital in 2015 might have lost genes over time.

The IncN plasmids pBHKPC93_3 and pBHKPC104_3 also have high similarity (99.96% identity and 100% coverage) with the IncN-pST15 plasmid, which has been circulating in different hosts [36]. We also compared these plasmids with pIncN_C1-94_KPC from Colombia [37], classified as part of an IncN “promiscuous” plasmids group that harbors the *bla*_KPC-2_ gene, and our analysis showed 97.7% identity and 81% coverage with pBHKPC93_3 and 99.24% identity and 81% coverage with pBHKPC104_3.

### 2.4. The Impact of bla_KPC_ Plasmid Presence in E. coli J53

In vitro conjugation assays using strains BHKPC93 and BHKPC104 as donors and *E. coli* J53 as an acceptor resulted in the transfer of only pBHKPC93_3 and pBHKPC104_3 plasmids with conjugation rates of approximately 2 × 10^−5^ transconjugants/recipient (Table 2).

S1 nuclease/PFGE data confirmed this conjugation, as only one ~50 kbp fragment was present in the *E. coli* J53 transconjugants originating from the donors BHKPC93 and BHKPC104. Despite the nonidentification of some plasmids using this technique, the presence of the six plasmids was evident from the hybrid assembly, considered a gold standard in this case, in BHKPC93 and BHKPC104 (Figure S6—Supplementary Materials).

The similar doubling times of *E. coli* J53 and the *E. coli* J53 transconjugants indicates that the plasmid acquisition did not seem to affect *E. coli* J53’s fitness in the absence of antibiotics (Table 2), with no statistically significant difference between *E. coli* J53 and the transconjugants *E. coli* J53 pBHKPC93 (*p* = 0.9790) and *E. coli* J53 pBHKPC104 (*p* = 0.9494).

The acquisition of pBHKPC93_3 and pBHKPC104_3 carrying the *bla*_KPC_ gene by *E. coli* J53 transconjugants resulted in a substantial increase in the meropenem and imipenem MICs. However, according to EUCAST criteria, these transconjugants are still susceptible (Table 2). 

The plasmid copy numbers were determined relatively with the values of a housekeeping/one copy chromosomic gene (*mdh*) and the *bla*_KPC_ or *bla*_NDM_ gene. The relative plasmid copy numbers (PCNs) revealed that the PCNs of the *bla*_KPC_-containing plasmids in *K. pneumoniae* were higher than in their *E. coli* transconjugants (Table 2). In addition, *bla*_KPC_-containing plasmids’ PCNs were higher than the *bla*_NDM_-containing plasmids’ PCNs in the BHKPC93 and BHKPC104 strains (Table 2).

## 3. Discussion

Carbapenems are last-resort antibiotics, and their use is increasing due to the difficulty of treating infections caused by multidrug-resistant *Enterobacterales*. This has led to an increase in carbapenem-resistant clinical isolates, becoming a threat to global public health due to the limited treatment options [38,39]. An even worse scenario occurs when carbapenemases are co-produced, such as KPC and NDM [39].

In Latin America, the first detection of isolates co-producing KPC and NDM was in *Enterobacter sp.* isolates from September/October 2013 [38,40]. Pereira et al. (2015) described a multidrug-resistant isolate, susceptible to amikacin and polymyxin B, carrying *bla*_KPC_ associated with Tn*4401b* in a 50 kb IncN plasmid and *bla*_NDM_ in a 160 kbp IncA/C plasmid associated to the ΔIS*Aba125* and *ble* genes. Both genes are associated with mobile genetic elements of worldwide epidemiological importance. 

Since its first detection, Argentina, Uruguay, Ecuador, Guatemala, and Paraguay have described isolates co-producing KPC and NDM at increasing rates between 2020 and 2021 [20]. Several studies have described this combination in Brazil since 2015, but so far neither isolates nor plasmids combining the KPC and NDM types of resistance have not been characterized in great detail [13,40,41,42].

In this study, we molecularly investigated a multidrug-resistant *K. pneumoniae* ST11 clone harboring six plasmids and 20 resistance genes, including *bla*_KPC-2_ and *bla*_NDM-1_, and characterized the plasmids containing carbapenemases genes.

The carbapenemases genes of this outbreak’s isolates were present in different plasmid groups than those identified in other Latin American countries. The IncA/C-type plasmids carrying *bla*_NDM-1_ were previously detected in *Enterobacterales* isolates in Brazil [40]. The IncA/C plasmids are associated with the dissemination of *bla*_NDM_, and these plasmids carrying the carbapenemase gene were isolated from lineages in many countries, such as Brazil, Germany, Romania, Iran, and Pakistan. They are usually larger than pBHKPC93_5 and pBHKPC104_5 and are widely spread [40,43,44,45,46,47,48].

*bla*_KPC_-harboring IncN plasmids, such as those identified in BHKPC93 and BHKPC104, were already considered “promiscuous” plasmids and had been detected in Brazil and Latin America in different STs and species, and these isolates had been detected in different hosts (human and dog) [36,37] (Figure S7—Supplementary Materials). Gomez-Simmonds et al. (2022) studied IncN plasmids harboring *bla*_KPC_ genes from a New York City Medical Centre and suggested evidence for their multispecies horizontal transmission [49]. *bla*_KPC_ IncN plasmids are widely spread, and they had already been found in isolates from different places, such as Brazil, Colombia, USA, Germany, Italy, and Israel [37,40,49,50,51,52,53,54,55,56]. Our analysis shows that a similar IncN plasmid harboring *bla*_KPC_ was already circulating within the same hospital, in the BHKPC49 strain (access number: SAMN32643894), where BHKPC93 and BHKPC104 were recovered in 2015, as a larger element of *K. pneumoniae* and which might have lost genes over time.

In the conditions analyzed in this study, the copy numbers of the *bla*_KPC_ plasmids in BHKPC93 and BHKPC104 were 11-fold and 4-fold higher than the plasmids containing *bla*_NDM_, which may have contributed to the fact that only the *bla*_KPC_ plasmids were conjugated to *E. coli* J53. According to EUCAST, *E. coli* J53 transconjugants are susceptible to these carbapenems, even though they are carbapenemase producers, which could contribute to a “silent” dissemination of the antimicrobial resistance gene. The lower copy numbers of the *bla*_KPC_ plasmids in the *E. coli* J53 transconjugants could explain the lower MIC values compared with *K. pneumoniae* BHKPC93 and BHKPC104. According to other studies, different plasmids in a bacterial cell can interact and improve fitness, replication, and gene horizontal transference [57,58]. The strains examined in this study harbor six plasmids, five of which carry toxin–antitoxin (T/A) systems, presumably contributing to their maintenance in bacteria. The small plasmids pBHKPC93_1 and pBHKPC104_1, carrying only a T/A system, could be advantageous, because they could allow cells to exchange plasmids without affecting viability. Studies show the association of a toxin family with different antitoxin families, suggesting that the TA system families’ concept is not adequate, opening the possibility that known toxin genes might be associated with genes representing novel antitoxins and vice versa [59,60,61]. The antitoxin HigA, encoded by these small plasmids, may complement antitoxins of plasmids with the same T/A system that could be lost, such as pBHKPC93_6 and pBHKPC104_6, which are larger and more costly to the cell. Only two plasmids, 1 and 2, were not considered self-conjugative. Plasmid 2, considered mobilizable, probably needs to share conjugative proteins codified by the plasmids in the cell to transfer. Despite the potential of being mobilized, only one plasmid was conjugated to *E. coli* J53. The PCN of the *bla*_KPC_-harboring plasmid were 11-fold and 9-fold higher in *K. pneumoniae* than in *E. coli*. So, in addition to the presence of other resistance genes, including β-lactamases, and mutation in porin-coding genes in *K. pneumoniae*, the interaction among different plasmids might play a role in the difference in the PCNs and carbapenem’s susceptibility in *E. coli* and *K. pneumoniae*, which deserves further investigation.

This study’s limitations include the lack of an analysis of the first isolates co-producing KPC/NDM found in the hospital because they were not stored. In addition, we could neither sequence the whole genome of all four isolates initially selected for the study nor the plasmids transferred to *E. coli* J53. We only checked the transconjugant *E. coli* plasmid sizes by S1 nuclease assay. Another limitation of this study is that with this method, we were unable to see whether the smaller plasmids were conjugated. Both plasmids, ~4.5 kb and ~5.2 kb, had no resistance genes, and the effect of their presence will be further analyzed by our group.

In conclusion, an extensively multidrug-resistant *K. pneumoniae* ST11 clone, co-producing KPC and NDM, caused the outbreak in a Brazilian teaching hospital during the COVID-19 pandemic. The *bla*_KPC_-harboring IncN plasmid shares a high identity with other plasmids from Latin America and was already circulating in 2015 as a larger element in the same hospital via the BHKPC49 strain (access number: SAMN32643894). The *bla*_KPC_ plasmid’s high copy number might have contributed to the conjugation of this gene to *E. coli*. *E. coli’s* susceptibility to meropenem and imipenem might be due to the fact of this species’ low copy number of the *bla*_KPC_ -harboring plasmid.

## 4. Materials and Methods

### 4.1. Bacterial Isolates

From October 2020 to January 2021, the hospital investigated 45 meropenem-resistant isolates, among which 30 were KPC producers, 6 NDM producers, and 6 KPC/NDM co-producers (Camila da Mata and Edna Leite, personal communication). Only four out of 6 KPC/NDM co-producers isolated from three patients were stored and included in this study: *K. pneumoniae* strains BHKPC93, BHKPC104, BHKPC107a, and BHKPC107b.

In addition, the hospital provided the isolate *K. pneumoniae* BHKPC49 from 2015, which has the *bla*_KPC-2_ gene and belongs to the hospital’s bacteria collection, which was already sequenced. We included BHKPC49 in this study to perform whole genome sequencing (WGS) and, therefore, characterize and compare the *bla*_KPC_ environment and plasmids to the outbreak isolates’ plasmids. All bacteria isolates were registered at SISGen under the number A6594DF.

### 4.2. Antimicrobial Susceptibility Test

The susceptibility profiles of all clinical isolates were determined using the Vitek-2 (Biomerieux, Marcy-l’Étoile, France) and CLSI 2020 recommendations by the hospital laboratory, except for meropenem, imipenem, colistin, polymyxin B, and tigecycline for which the minimal inhibitory concentrations (MICs) were determined at the LEMiMo/University of São Paulo, along with the transconjugant strains, using the microdilution method according to the ISO 20776-1 and EUCAST recommendations.

### 4.3. Genomic DNA Extractions

For polymerase chain reactions (PCRs), the bacteria were inoculated in brain heart infusion (BHI) broth (Kasvi, Parana, Brazil), and the cells were lysed with the mechanical lysis method using glass beads [62]. Nucleic acids were purified by precipitation in isopropanol, rehydrated in ultrapure water, and treated with RNase. The extracted DNA was quantified in a NanoDrop ND 2000c (Thermofisher, Waltham, MA, USA) and stored at −20 °C.

For the genome sequencing, bacterial DNA was extracted according to the manufacturer’s instructions using the Wizard Genomic DNA Purification Kit (Promega, Madison, WI, USA). The extracted DNA was quantified using a NanoDrop ND 2000c and stored at 4 °C.

### 4.4. bla_KPC_ and bla_NDM_ Genes and Tn4401 Detection

The *bla*_KPC_ and *bla*_NDM_ genes were detected individually using PCR with primers described by Poirel et al. (2011) [63,64]. The amplification of Tn*4401* was performed using the primer pairs described by Naas et al. (2008) (Table S1—Supplementary Materials) [64].

According to the manufacturer’s instructions, the reactions were prepared using Taq DNA polymerase, recombinant (Thermo Scientific, Vilnius, Lithuania). The PCRs were performed for 35 cycles at 95 °C for 30 s, 55 °C for 30 s, and 72 °C for 60 s. The PCR products were verified with 1% agarose gel electrophoresis at 90 V for 40 min (PowerPac™ HC High-Current Power Supply, Bio-Rad, Hercules, CA, USA).

### 4.5. Detection of the Clonality and Plasmid Sizes

The clonality and plasmid sizes were detected after XbaI and S1-nuclease restriction, respectively, followed by pulsed-field gel electrophoresis (PFGE). Two milliliters of overnight cultures adjusted for OD_600nm_ 1 were used to separate and wash the cells. Agarose plugs were made with 400 µL of cell suspension, 40 µL of proteinase K (10 mg/mL), and 400 µL of 1.25% Seakem Gold Agarose (Lonza, Basel, Switzerland). The immobilized cells in the plugs were lysed using 50 mM Tris-HCl pH8, 50 mM EDTA pH8, 1% Sarcosil, and 50 µL of proteinase K (10 mg/mL) for 2 h at 55 °C. The plugs were then washed with Tris-EDTA buffer and ultrapure water.

According to the manufacturer’s instructions, 5 × 5 mm plug fragments were digested with XbaI or S1-nuclease and submitted to electrophoresis in 1% Seakem Gold Agarose (Lonza, Basel, Switzerland). XbaI-PFGE gel’s run conditions: 6 V gradient, 19 h, 6.76 s initial switch time, and 35.35 s final switch time using the CHEF Mapper system (Bio-Rad, Hercules, CA, USA). A Lambda DNA Ladder (48.5 KB–1 MB) (Lonza, Basel, Switzerland) was used as the molecular weight standard. S1-nuclease PFGE gel’s run conditions: 6 V gradient, 15 h, 1 s initial switch time, and 18 s final switch time. Low Range PFG Marker (New England Biolabs, Ipswich, MA, USA) was used as the molecular weight standard.

The gels were dyed with SYBR Safe DNA Gel Stain (Thermo Fisher Scientific, Waltham, MA, USA) for 60 min and revealed using the ChemiDoc MP Imaging System (Bio-Rad, Hercules, CA, USA). The PFGE gel image was analyzed using Bionumerics software v. 7.1 (Applied Maths, Sint-Martens-Latem, Belgium) to determine the genetic similarity among the isolates.

### 4.6. Determination of the Mucoid Phenotypes 

The mucoid phenotypes were determined using the String test, performed as described elsewhere [65,66]. The isolates’ colonies were grown on BHI and MacConkey Agar (Kasvi, Brazil) plates and touched with an inoculation loop. The loop was lifted, and if a string longer than 5 mm was observed, the strain was classified as hypermucoviscous. Otherwise, the strains were classified as mucoid.

### 4.7. Genome Sequencing

The genomes were sequenced using the Illumina and Nanopore methods at the University Medical Centre Utrecht, Utrecht (The Netherlands). For the Illumina sequencing, the library was prepared with the Nextera DNA sample preparation kit and Nextera index v2 set D for 96 indexes, and the isolates were sequenced using NextSeq500 2 × 150 bp mid-output (120 M clusters) (Illumina, San Diego, CA, USA).

For the Nanopore sequencing, the libraries were prepared using Oxford Nanopore’s Ligation Sequencing Kit and Native Barcoding Expansion Kit, and the isolates were sequenced using the MinION sequencer (Oxford Nanopore Technologies, Oxford, UK).

### 4.8. Quality Analysis and Genome Assembly

Initial quality analysis of Illumina’s reads was performed using Fastqc (v0.11.9). Trim Galore (v.0.6.6) with the default parameters was applied for adapter removal and quality trimming. The genomes were assembled using Unicycler (v.0.4.8) in the bold mode. The hybrid assemblies’ quality was assessed using BUSCO (v5.1.2).

The assembled BHKPC93, BHKPC104, and BHKPC49 genomes were submitted to the NCBI database for annotation and deposited with the accession numbers SAMN26563515, SAMN26563478, and SAMN32643894, respectively.

The genome sequences were analyzed using Resfinder, MLST, and VirulenceFinder, available at the Center for Genomic Epidemiology (http://www.genomicepidemiology.org/ accessed on 8 March 2023).

### 4.9. Genomes Similarity Analysis

The number of SNPs among the isolates was determined using the CFSAN pipeline (v. 2.1.1) with the default parameters. For this, the Illumina reads of isolate BHKPC93 were mapped against the assembled genome of BHKPC104.

The genomes were annotated with BAKTA (v1.3.3), and the core and accessory genes were identified using Panaroo (v1.2.10) with the default parameters. The gene content similarity was evaluated by calculating the Jaccard distance between the isolates, as indicated below:

$$Jaccarddistance=1-\frac{\left|GenesBHKPC93\cap GenesBHKPC104\right|}{\left|GenesBHKPC93\cup GenesBHKPC104\right|}$$

The plasmids sequences were submitted to NCBI BLASTn, and Kablammo software with the default parameters was used to analyze the plasmids’ identity and compare with BHKPC49 (accession number: SAMN32643894), IncN-pST15 plasmids (accession numbers: JABSUB010000003.1, CP004367.2, and KX062091.1) [36,37], and pIncN_C1-94_KPC from Colombia [37].

### 4.10. Code Availability

The codes to reproduce the bioinformatic analysis of this work are publicly available: gitlab.com/jpaganini/saopaulo_kpn_outbreak.

### 4.11. Multilocus Sequence Typing (MLST)

The STs of the four isolates were determined according to the MLST Pasteur scheme [67]. For BHKPC93 and BHKPC104, the whole genome sequence data were used. For BHKPC107a and BHKPC107b, which were not submitted for WGS, the PCRs were conducted with specific MLST *K. pneumoniae* primers [67], and the amplicons were sequenced using the Sanger method, carried out on an Applied Biosystems 3130 Genetic Analyzer (Life Technologies, Carlsbad, CA, USA).

### 4.12. Plasmids Analysis

The plasmids’ mobilization capacity was determined using Plascad software with default parameters.

The genes of the toxin/antitoxin (T/A) systems were detected using the TA Finder web service (https://bioinfo-mml.sjtu.edu.cn/TADB2/index.php accessed on 8 March 2023) with the default parameters.

### 4.13. Conjugation Assay

Overnight cultures of the receptor strain *E. coli* J53 and the donor strains *K. pneumoniae* BHKPC93 and *K. pneumoniae* BHKPC104 were adjusted to OD_600nm_ 0.3 in 10 mL lysogeny broth (LB) (Kasvi, Brazil) and incubated at 37 °C until OD_600nm_ 0.7. The conjugation assays were performed in 10 mL of 1:1 (receptor:donor) OD_600nm_ 0.7 cultures, which were incubated at 37 °C for 24 h. Serial dilutions of the co-cultures were spread on LB agar with 125 mg/L azide and LB agar with 4 mg/L imipenem and 125 mg/L azide. After incubation, the isolated *E. coli* colonies were counted, and the conjugation rate was calculated using (CFU_receptor_/mL)/(CFU_transconjugant_/mL). The isolated colonies detected on imipenem- and azide-containing LB agar were subjected to PCR to detect the *bla*_KPC_ and *bla*_NDM_ genes using the same culture suspension as the templates.

### 4.14. Determination of the Growth Curve and Doubling Time 

The overnight cultures were adjusted to OD_600nm_ 0.05–0.1 in cation-adjusted Mueller Hinton broth ( Becton Dickinson and Company, Sparks, MD, USA), and 200 µL of each were added to each well in a 96-well polystyrene microplate. The OD_600nm_ measurements were taken every 15 min with 5 s of agitation before and after the reading using the SpectraMax M5 spectrophotometer (Molecular Devices, San Jose, CA, USA) at 37 °C for 10 h. The assay was performed in five replicates. The measurements were plotted on a growth curve, and the doubling times were calculated using linear regression of the log-phase. The statistical significance was calculated using the Student’s t-test and ANOVA (*p* < 0.05).

### 4.15. Determination of the Plasmid Copy Number 

The plasmid copy numbers were determined using qPCR, according to the method described by Woodall, C. A. (2003) [68]. The overnight cultures were adjusted to OD_600nm_ 1 in BHI broth (Kasvi, Parana, Brazil), and the cells were lysed at 98 °C for 10 min, followed by freezing for 10 min at 20 °C. The lysed cultures were diluted at 1:10, and 3 µL were used in each reaction. The primers were designed for the housekeeping genes (malate dehydrogenase (*mdh*) genes) and *bla*_KPC_/*bla*_NDM_ genes. The qPCR reactions contained 1x SYBR Green qPCR Master Mix (ThermoFisher, Waltham, CA, USA), 100 nM of each primer, 3 µL of culture, and ultrapure water qs to 10 µL, and they were conducted using the CFX96 Touch Real-Time PCR Detection System (Bio-Rad, Hercules, CA, USA) with the following conditions: 3 min at 95 °C, 40 cycles of 10 s at 95 °C, 1 min at 50 °C, and 10 s at 72 °C. All qPCR reactions, including the controls, were performed in experimental triplicates. The primers’ efficiency and specificity were determined using standard and melting curves, with five serial dilutions. The standard curves’ slopes were used to calculate the amplification efficiency [69].

The relative plasmid copy numbers (PCNs) were determined, considering the different amplification efficiencies and Ct values for each amplicon (chromosomic -c and plasmid -p) [69].

## Figures and Tables

**Table 1:** General data and susceptibility profile of the isolates.

	BHKPC93		BHKPC104		BHKPC107a		BHKPC107b	
**Isolates’ general data**								
Species	*K. pneumoniae*		*K. pneumoniae*		*K. pneumoniae*		*K. pneumoniae*	
Isolation date (DD/MM/YYYY)	11/12/2020		30/12/2020		01/01/2021		07/01/2021	
Clinical specimen	Catheter tip		Urine		Deep tissue		Deep tissue	
**Antibiotics tested (susceptibility/MIC)**								
Nalidixic Acid	-	-	R	-	-	-	-	-
Amikacin	S	-	S	-	S	-	S	-
Amoxicillin/Clavulanic Acid	R	-	R	-	R	-	R	-
Ampicillin/Sulbactam	R	-	R	-	R	-	R	-
Cefepime	R	-	R	-	R	-	R	-
Ceftazidime	R	-	R	-	R	-	R	-
Ceftriaxone	R	-	R	-	R	-	R	-
Cefuroxime	R	-	R	-	R	-	R	-
Axetil Cefuroxime	R	-	R	-	R	-	R	-
Ciprofloxacin	R	-	R	-	R	-	R	-
Colistin	R	16 mg/L	R	16 mg/L	R	16 mg/L	R	16 mg/L
Ertapenem	R	-	R	-	R	-	R	-
Gentamicin	R	-	R	-	R	-	S	-
Imipenem	R	64 mg/L	R	128 mg/L	R	64 mg/L	R	64 mg/L
Meropenem	R	128 mg/L	R	256 mg/L	R	256 mg/L	R	256 mg/L
Nitrofurantoin	-	-	R	-	-	-	-	-
Norfloxacin	-	-	R	-	-	-	-	-
Piperacillin/Tazobactam	R	-	R	-	R	-	R	-
Polymyxin B	R	8 mg/L	R	8 mg/L	R	8 mg/L	R	8 mg/L
Tigecycline	-	1 mg/L	-	2 mg/L	-	1 mg/L	-	0.5 mg/L
Trimethoprim/Sulfamethoxazole	R	-	R	-	R	-	R	-

R, resistant; S, susceptible; -, not determined.

**Table 2:** Characterization data of *bla*_KPC_ and *bla*_NDM_ plasmids.

	Conjugation Rate	Doubling Time (min)	Imipenem MIC (mg/L)	Meropenem MIC (mg/L)	PCN (*bla*_KPC_ Plasmid/*bla*_NDM_ Plasmid)
*E. coli* J53	-	111 ± 16	0.125	0.0625	-/-
*K. pneumoniae* BHKPC93	-	-	64	128	20.1 ± 0.1/1.86 ± 0.1
*K. pneumoniae* BHKPC104	-	-	128	256	7.14 ± 0.03/1.85 ± 0.07
*E. coli* J53 + pBHKPC93_3	2.1 × 10^−5^	111 ± 13	2	2	1.87 ± 0.05/-
*E. coli* J53 + pBHKPC104_3	1.7 × 10^−5^	112 ± 14	2	2	0.78 ± 0.02/-

## Data Availability

The data presented in this study are available upon request from the corresponding author.

## Supplementary Materials

The following supporting information can be downloaded at: https://www.mdpi.com/article/10.3390/antibiotics12050926/s1, Table S1: Sequences of primers used in this study; Table S2: Description of the 15 and 11 genes found exclusively in BHKPC93 and BHKPC104; Table S3: Resistance genes found in BHKPC93 and BHKPC104; Figure S1: Scheme of the chromosome comparison using BHKPC104 as a reference and compared with BHKPC93; Figure S2: Scheme of pBHKPC93_3; Figure S3: Scheme of pBHKPC104_3; Figure S4: Scheme of pBHKPC93_5; Figure S5: Scheme of pBHKPC104_5; Figure S6: S1 nuclease PFGE gel of the isolates BHKPC93 (A), BHKPC104 (B), BHKPC107a (C), and BHKPC107b (D) and the transconjugants J53_pBHKPC93_3 (E) and J53_pBHKPC104_3 (F); Figure S7: Scheme of the comparison between pBHKPC93_3 and pBHKPC104_3.
