# Supplementary material for: Characterization of blaKPC-2 and blaNDM-1 Plasmids of a K. pneumoniae ST11 Outbreak Clone
Source: Antibiotics (Basel). 2023 May 18;12(5):926. doi: 10.3390/antibiotics12050926 (PMC10215860; doi:10.3390/antibiotics12050926)
Supplement: Supplementary file 1 [file antibiotics-12-00926-s001.zip › antibiotics-2370589-supplementary.pdf]

Table S1. Sequences of primers used in this study.

Primer	Sequence (5′ – 3′)
*bla*KPC_F	CGTCTAGTTCTGCTGTCTTG
*bla*KPC_R	CTTGTCATCCTTGTTAGGCG
*bla*NDM_F	GGTTTGGCGATCTGGTTTTC
*bla*NDM_R	CGGAATGGCTCATCACGATC
4281	GGCACGGCAAATGACTA
4714	GAAGATGCCAAGGTCAATGC
3781L	GCTTTCTTGCTGCCGCTGTG
3098U	TGACCCTGAGCGGCGAAAGC
905L	GCGACCGGTCAGTTCCTTCT
816U	CACCTACACCACGACGAACC
*mdh_Ecoli*_F	CGATCTGAGCCATATCCCTACT
*mdh_Ecoli*_R	GAACGATCCATACCCGGTTTAC
*mdh_Kpneumoniae*_F	GCGGATGTAGTGCTGATCTC
*mdh_Kpneumoniae*_R	CTGCTGCACGAGGTTCTT
*bla*KPC_qPCR_F	GGCGGCTCCATCGGTGTGTA
*bla*KPC_qPCR_R	AATTGGCGGCGGCGTTATCA
*bla*NDM_qPCR_F	GATTGCGACTTATGCCAATG
*bla*NDM_qPCR_R	TCGATCCCAACGGTGATATT

Table S2. Description of the 15 and 11 genes found exclusively in BHKPC93 and BHKPC104.

Proteins of unique genes	Present only in	Chromosome/plasmid	Contig / Coordinate / Strand
IS6-like element IS26 family transposase	BHKPC93	Plasmid pBHKPC93_4	5 / 51764 – 52468 / -
IS6-like element IS26 family transposase	BHKPC93	Plasmid pBHKPC93_4	5 / 48769 – 49473 / +
IS6-like element IS26 family transposase	BHKPC93	Plasmid pBHKPC93_6	3 / 83846 – 84550 / +
Tn3-like element Tn5403 family transposase	BHKPC93	Plasmid pBHKPC93_6	3 / 81730 – 83787 / -
IS3 -like element ISKpn18 family transposase	BHKPC93	Plasmid pBHKPC93_5	4 / 89237 – 90115 / +
Cobalamin biosynthesis protein CbiX	BHKPC93	Plasmid pBHKPC93_3	6 / 31925 – 32205 / +
Cobalamin biosynthesis protein CbiX	BHKPC93	Plasmid pBHKPC93_3	6 / 32630 - 32926/ +
ANT(3'')-Ia family aminoglycoside nucleotidyltransferase AadA2	BHKPC93	Plasmid pBHKPC93_6	3 / 79338 – 80117 / -
quaternary ammonium compound efflux SMR transporter QacE delta 1	BHKPC93	Plasmid pBHKPC93_6	3 / 78827 – 79174 / -
sulfonamide-resistant dihydropteroate synthase Sul1	BHKPC93	Plasmid pBHKPC93_6	3 / 77994 – 78833 / -
hypothetical protein	BHKPC93	Plasmid pBHKPC93_3	6 / 31380 – 31712 / +
hypothetical protein	BHKPC93	Plasmid pBHKPC93_3	6 / 32274 – 32474 / +
hypothetical protein	BHKPC93	Plasmid pBHKPC93_3	6 / 32959 – 33066 / +
hypothetical protein	BHKPC93	Plasmid pBHKPC93_6	3 / 77594 – 77866 / -
MFS-type transporter	BHKPC93	Chromosome	1 / 1497284 – 1498471 / -
IS6-like element IS26 family transposase	BHKPC104	Plasmid pBHKPC104_6	2 / 164221 – 164826 / +
Anti-restriction protein ArdA	BHKPC104	Plasmid pBHKPC104_3	5 / 36942 – 37451 / +
Anti-restriction protein ArdA	BHKPC104	Plasmid pBHKPC104_3	5 / 35600 – 36109 / +
TrbG/VirB9 family P-type conjugative transfer protein	BHKPC104	Plasmid pBHKPC104_5	3 / 27981 – 28421 / +
aminoglycoside O-phosphotransferase APH(3')-Ia	BHKPC104	Plasmid pBHKPC104_6	2 / 165016 – 165831 / -
IS5-like element ISKpn13 family transposase	BHKPC104	Chromosome	1 / 2993526 – 2994460 / +
IS5-like element ISKpn26 family transposase	BHKPC104	Chromosome	1 / 2680867 – 2681847 / -
Carbohydrate porin	BHKPC104	Choromosome	1 / 4650295 – 4651677 / -
Carbohydrate porin	BHKPC104	Chromosome	1 / 50221 – 51600 / -
MFS transporter	BHKPC104	Chromosome	1 / 2710758 – 2710979 / +
hypothetical protein	BHKPC104	Chromosome	1 / 4692771 – 4692992 / +

TableS3. Resistance genes found in BHKPC93 and BHKPC104.

Resistance gene	Identity	Alignment Length/Gene Length	Position in reference	Phenotype	Accession no.
aac(3)-IIa	100.0	861/861	1..861	gentamicina, tobramycin,	CP023555
aac(6')-Ib-cr	100.0	600/600	1..600	ciprofloxacin	DQ303918
aph(3')-Ia	100.0	816/816	1..816	neomycin, kanamycin, lividomycin, paromomycin, ribostamycin	V00359
aadA2	100.0	792/792	1..792	spectinomycin, streptomycin	JQ364967
fosA	99.27	412/420	1..412	fosfomycin	ACWO0100 0079
sul1	100.0	840/840	1..840	sulfamethoxazole	U12338
dfrA12	100.0	498/498	1..498	trimethoprim	AM040708
OqxB	100.0	3153/3153	1..3153	chloramphenicol, benzylkonium chloride, cetylpyridinium chloride, nalidixic acid, ciprofloxacin, trimethoprim	EU370913
OqxA	100.0	1176/1176	1..1176	chloramphenicol, benzylkonium chloride, cetylpyridinium chloride, nalidixic acid, ciprofloxacin, trimethoprim	EU370913
sul2	100.0	816/816	1..816	sulfamethoxazole	AY034138
qnrS1	100.0	657/657	1..657	ciprofloxacin	AB187515
tet(A)	99.92	1200/1200	1..1200	doxycycline, tetracycline	AJ517790
$bla_{SHV-182}$	99,88	861/861	1..861	unknown beta-lactam	KP050489
bla_{KPC-2}	100.0	882/882	1..882	amoxicillin, amoxicillin+clavulanic acid, ampicillin, ampicillin+clavulanic acid, aztreonam, cefepime, cefotaxime, cefoxitin, ceftazidime, ertapenem, imipenem, meropenem, piperacillin, piperacillin+tazobactam, ticarcillin, ticarcillin+clavulanic acid	AY034847

bla$_{NDM-1}$	100.0	813/813	1..813	amoxicillin, amoxicillin+clavulanic acid, ampicillin, ampicillin+clavulanic acid, cefepime, cefixime, cefotaxime, cefoxitin, ceftazidime, ertapenem, imipenem, meropenem, piperacillin, piperacillin+tazobactam, temocillin	FN396876
bla$_{LAP-2}$	100.0	858/858	1..858	amoxicillin, ampicillin, cephalotin, piperacillin, ticarcillin	EU159120
bla$_{OXA-1}$	100.0	831/831	1..831	amoxicillin, amoxicillin+clavulanic acid, ampicillin, ampicillin+clavulanic acid, cefepime, piperacillin, piperacillin+tazobactam	HQ170510
bla$_{CTX-M-15}$	100.0	876/876	1..876	amoxicillin, ampicillin, aztreonam, cefepime, cefotaxime, ceftazidime, ceftriaxone, piperacillin, ticarcillin	AY044436
mph(A)	100.0	906/906	1..906	erythromycin, azithromycin, spiramycin, telithromycin	D16251
qacE	100.0	282/333	1..282	benzylkonium chloride, ethidium bromide, chlorhexidine, cetylpyridinium chloride	X68232
catB3	100.0	442/633	1..442	chloramphenicol	U13880

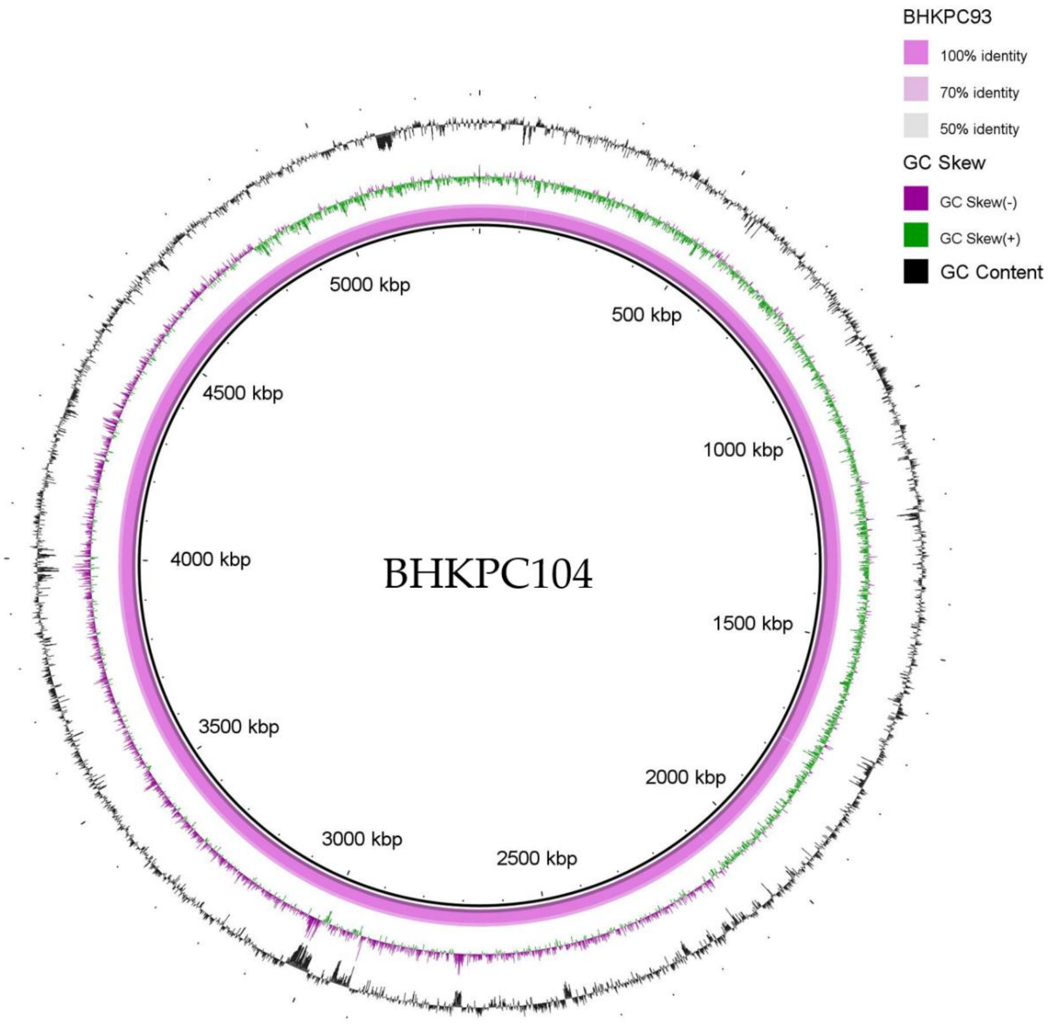

**Comparison**	BHKPC93xBHKPC104	BHKPC104xBHKPC93
**% of identity/coverage**	100/99	100/100

Figure S1. Scheme of chromosome comparison using BHKPC104 as reference and comparing with BHKPC93.

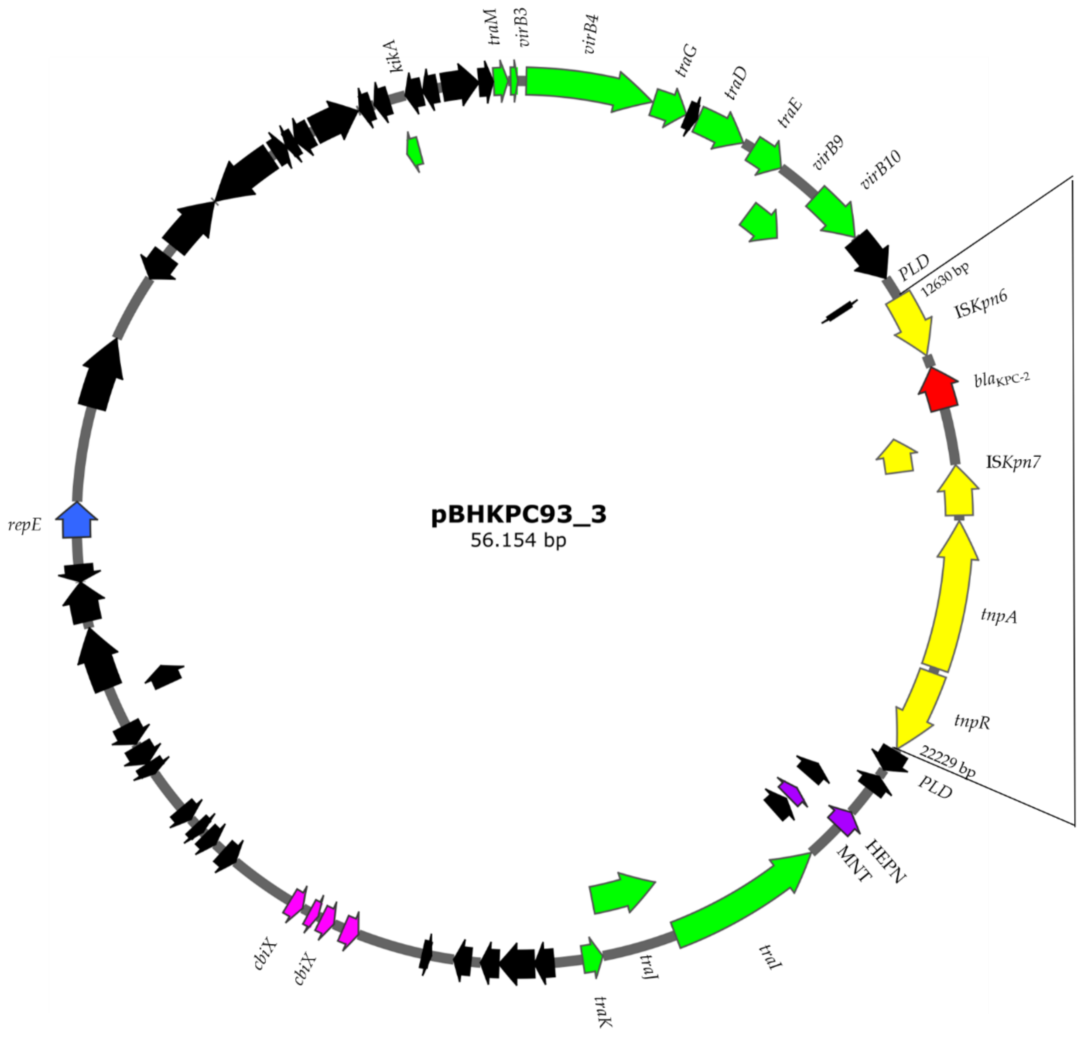

Figure S2. Scheme of pBHKPC93_3. Carbapenemase gene is represented in red, genetic environment genes of *bla*KPC are represented in yellow, plasmid replication initiator is represented in blue, T/A system genes are represented in purple, genes involved in plasmid conjugation are represented in green and the unique genes present only in BHKPC93 are represented in pink.

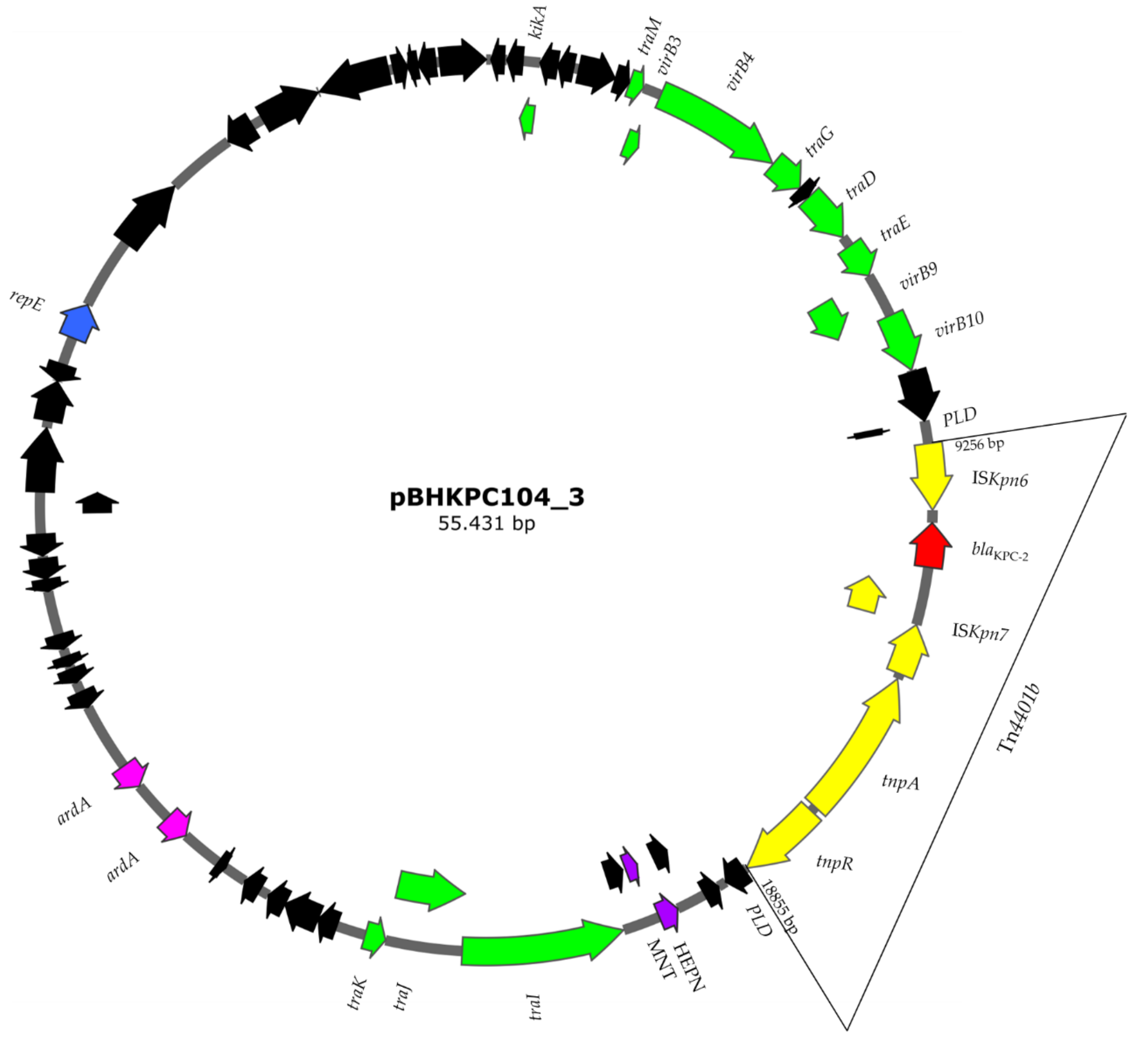

Figure S3. Scheme of pBHKPC104_3. Carbapenemase gene is represented in red, genetic environment genes of *bla*KPC are represented in yellow, plasmid replication initiator is represented in blue, T/A system genes are represented in purple, genes involved in plasmid conjugation are represented in green and the unique genes present only in BHKPC104 are represented in pink.

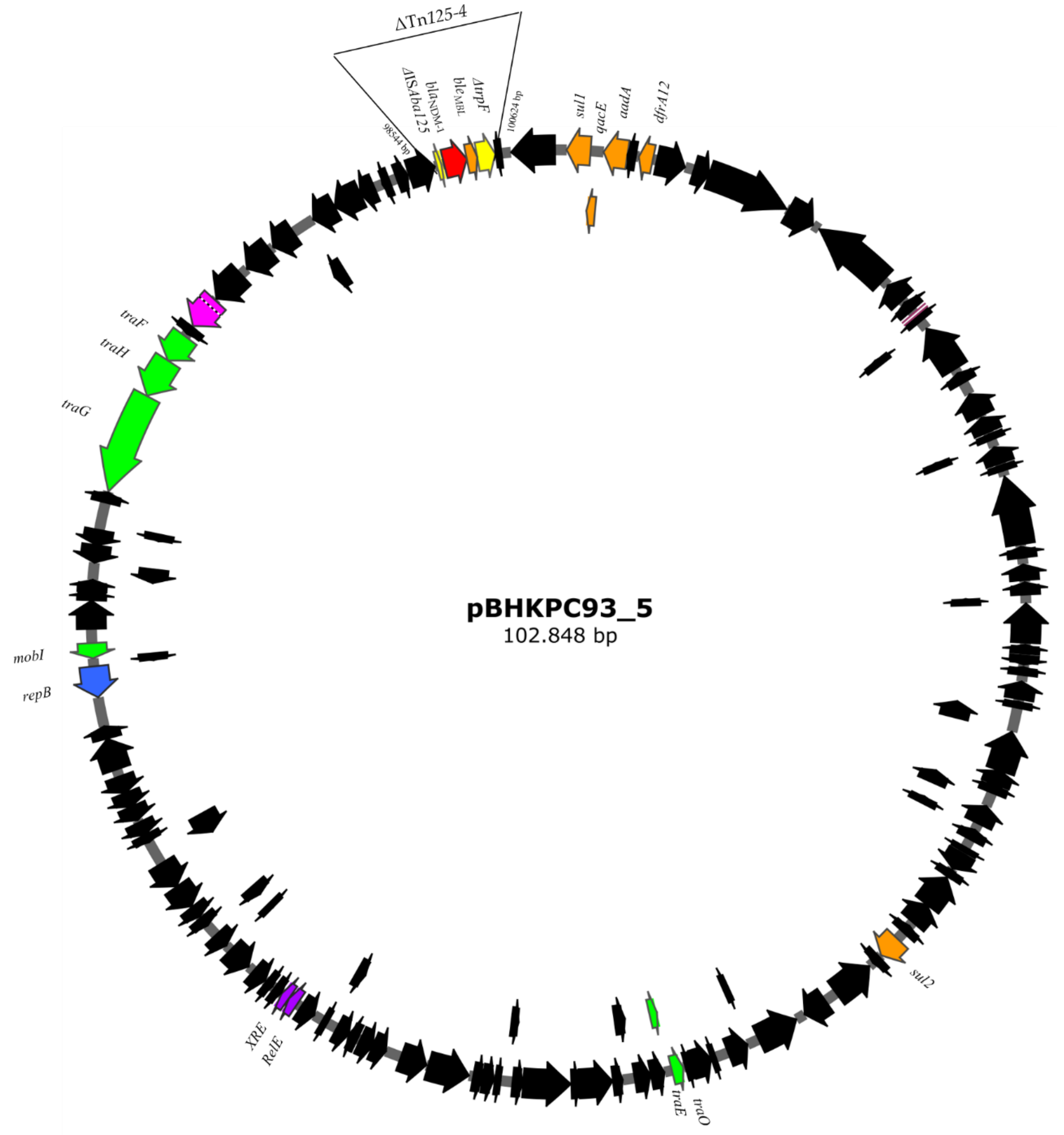

Figure S4. Scheme of pBHKPC93_5. Carbapenemase gene is represented in red, genetic environment genes of *bla*NDM are represented in yellow, other resistance genes are represented in Orange, plasmid replication initiator is represented in blue, T/A system genes are represented in purple, genes involved in plasmid conjugation are represented in green and the unique genes present only in BHKPC93 are represented in pink.

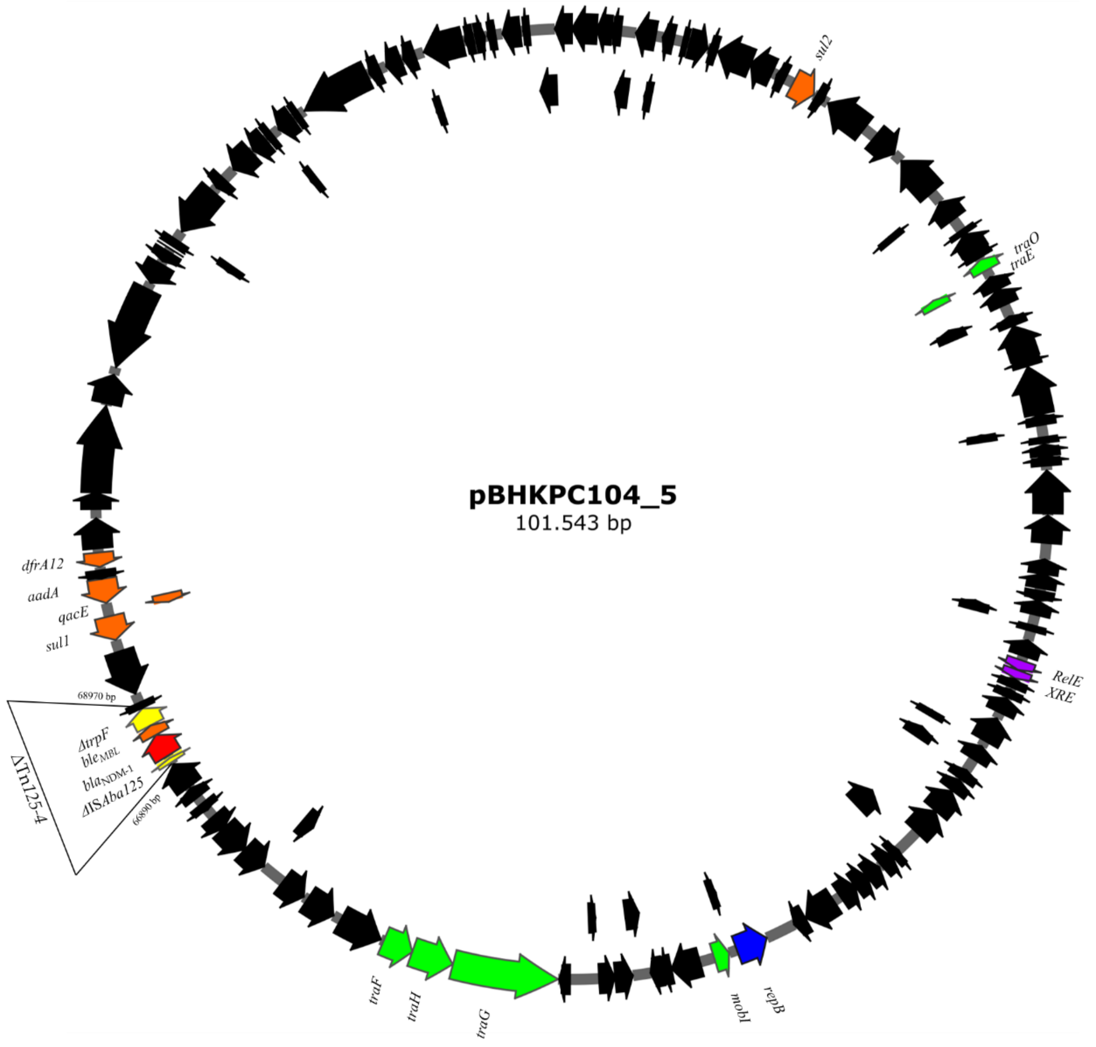

Figure S5. Scheme of pBHKPC104_5. Carbapenemase gene is represented in red, genetic environment genes of *bla*$_{NDM}$ are represented in yellow, other resistance genes are represented in Orange, plasmid replication initiator is represented in blue, T/A system genes are represented in purple and genes involved in plasmid conjugation are represented in green.

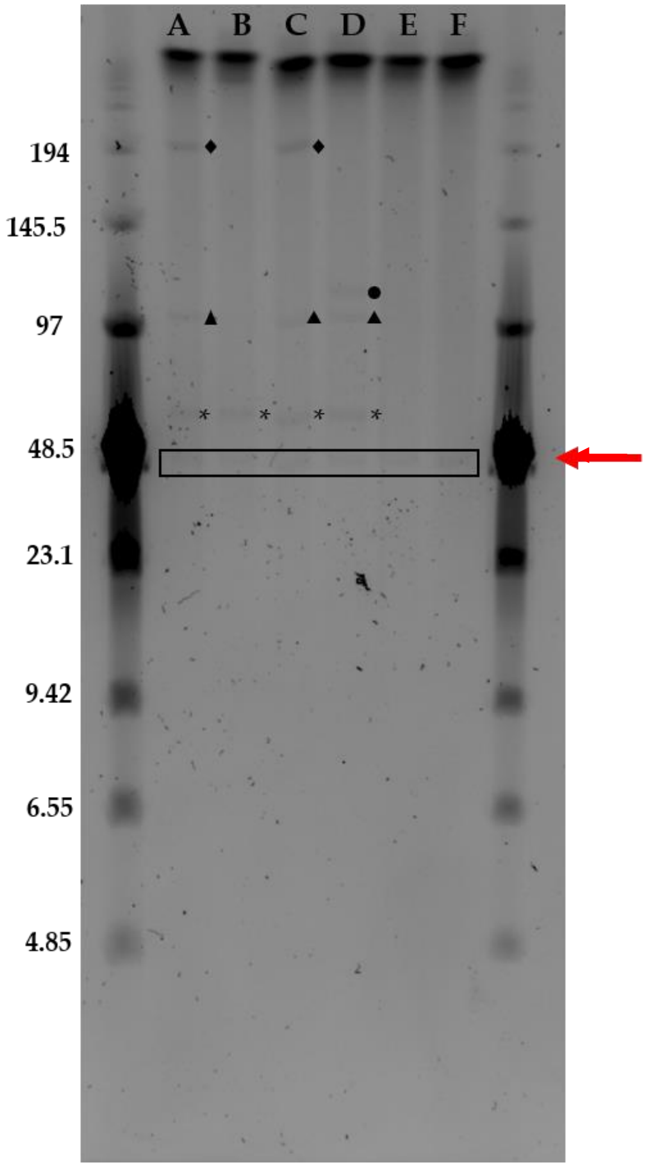

Figure S6. S1 nuclease PFGE gel of the isolates BHKPC93 (A), BHKPC104 (B), BHKPC107a (C), and BHKPC107b (D), and the transconjugants J53_pBHKPC93_3 (E) and J53_pBHKPC104_3 (F). Red arrow indicates the bands corresponding to the *bla*$_{kpc}$ plasmid. Rhombus, circle, triangles, asterisks and the rectangle highlight the plasmid bands. Low Range PFG Marker (New England Biolabs, EUA) was used as molecular weight and it has been run on both of the outer lanes.

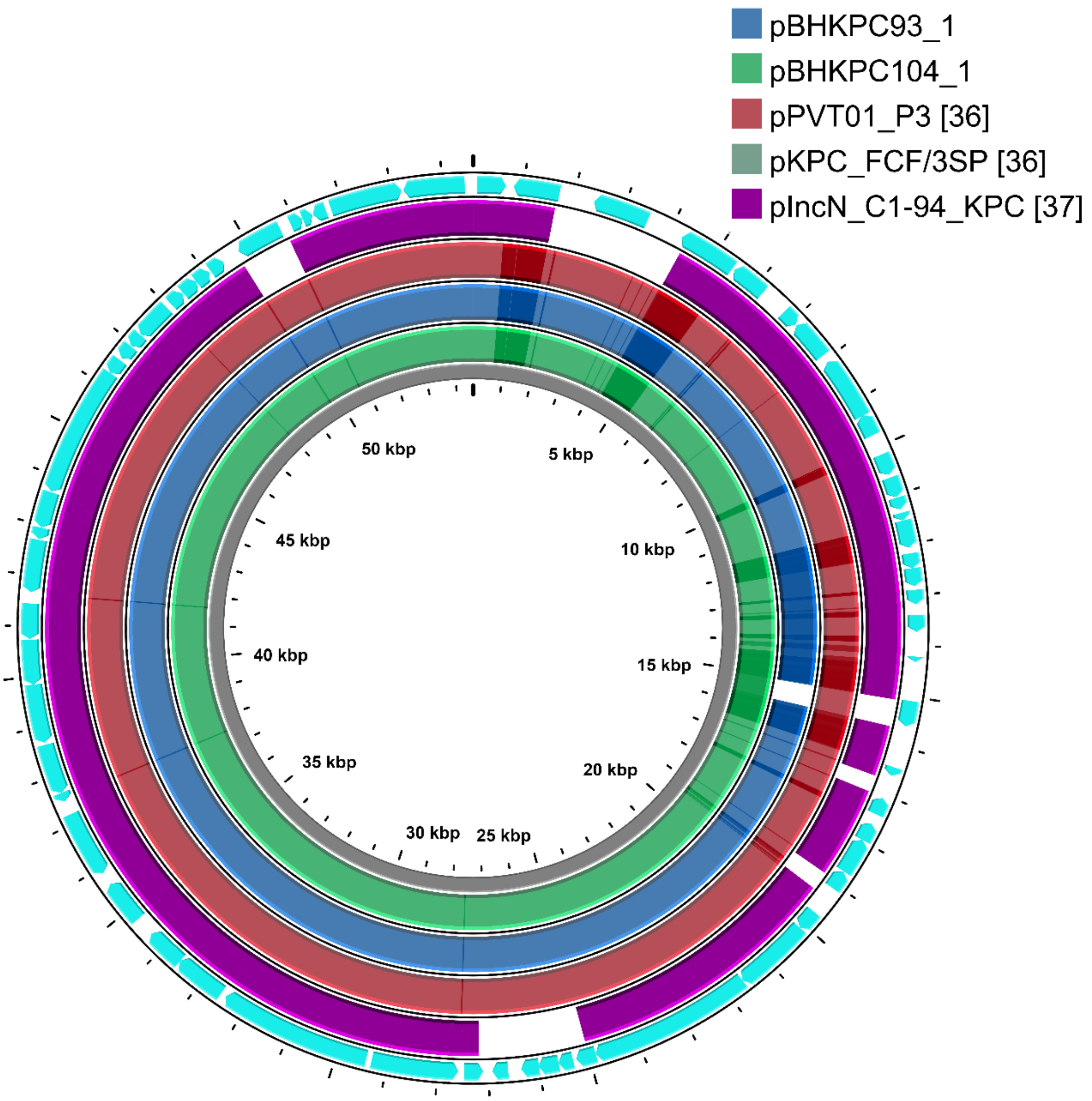

Figure S7. Scheme of the comparison between pBHKPC93_3 and pBHKPC104_3 with another IncN - plasmids harboring *bla*$_{KPC}$ already described.
